# Decreased expression of the NLRP6 inflammasome is associated with increased intestinal permeability and inflammation in obesity with type 2 diabetes

**DOI:** 10.1007/s00018-024-05124-3

**Published:** 2024-02-05

**Authors:** Gema Frühbeck, Javier Gómez-Ambrosi, Beatriz Ramírez, Sara Becerril, Amaia Rodríguez, Amaia Mentxaka, Víctor Valentí, Rafael Moncada, Gabriel Reina, Jorge Baixauli, Marcos Casado, Camilo Silva, Javier Escalada, Victoria Catalán

**Affiliations:** 1 Metabolic Research Laboratory, Clínica Universidad de Navarra, Avda. Pío XII, 36, 31008 Pamplona, Spain; 2 CIBER Fisiopatología de la Obesidad y Nutrición (CIBEROBN), Instituto de Salud Carlos III, Pamplona, Spain; 3 Obesity and Adipobiology Group, Instituto de Investigación Sanitaria de Navarra (IdiSNA), Pamplona, Spain; 4 Department of Endocrinology & Nutrition, Clínica Universidad de Navarra, Pamplona, Spain; 5 Department of Surgery, Clínica Universidad de Navarra, Pamplona, Spain; 6 Department of Anesthesia, Clínica Universidad de Navarra, Pamplona, Spain; 7 Department of Microbiology, Clínica Universidad de Navarra, Pamplona, Spain

**Keywords:** NLRP, Inflammasome, Inflammation, Obesity, Type 2 diabetes, Intestinal integrity, Jejunum

## Abstract

**Background:**

Obesity-associated dysfunctional intestinal permeability contributes to systemic chronic inflammation leading to the development of metabolic diseases. The inflammasomes constitute essential components in the regulation of intestinal homeostasis. We aimed to determine the impact of the inflammasomes in the regulation of gut barrier dysfunction and metabolic inflammation in the context of obesity and type 2 diabetes (T2D).

**Methods:**

Blood samples obtained from 80 volunteers (*n* = 20 normal weight, *n* = 21 OB without T2D, *n* = 39 OB with T2D) and a subgroup of jejunum samples were used in a case–control study. Circulating levels of intestinal damage markers and expression levels of inflammasomes as well as their main effectors (IL-1β and IL-18) and key inflammation-related genes were analyzed. The impact of inflammation-related factors, different metabolites and *Akkermansia muciniphila* in the regulation of inflammasomes and intestinal integrity genes was evaluated. The effect of blocking *NLRP6* by using siRNA in inflammation was also studied.

**Results:**

Increased circulating levels (*P* < 0.01) of the intestinal damage markers endotoxin, LBP, and zonulin in patients with obesity decreased (*P* < 0.05) after weight loss. Patients with obesity and T2D exhibited decreased (*P* < 0.05) jejunum gene expression levels of *NLRP6* and its main effector *IL18* together with increased (*P* < 0.05) mRNA levels of inflammatory markers. We further showed that while NLRP6 was primarily localized in goblet cells, NLRP3 was localized in the intestinal epithelial cells. Additionally, decreased (*P* < 0.05) mRNA levels of *Nlrp1*, *Nlrp3* and *Nlrp6* in the small intestinal tract obtained from rats with diet-induced obesity were found. *NLRP6* expression was regulated by taurine, parthenolide and *A. muciniphila* in the human enterocyte cell line CCL-241. Finally, a significant decrease (*P* < 0.01) in the expression and release of *MUC2* after the knockdown of *NLRP6* was observed.

**Conclusions:**

The increased levels of intestinal damage markers together with the downregulation of *NLRP6* and *IL18* in the jejunum in obesity-associated T2D suggest a defective inflammasome sensing, driving to an impaired epithelial intestinal barrier that may regulate the progression of multiple obesity-associated comorbidities.

**Supplementary Information:**

The online version contains supplementary material available at 10.1007/s00018-024-05124-3.

## Background

Chronic and unresolved inflammation is a hallmark of obesity that leads to the dysfunction of adipose tissue (AT), promoting the development of its associated comorbidities [1, 2]. Low-grade systemic inflammation involves an intricate network of pathways affecting not only the AT but also interconnecting metabolic organs including the gastrointestinal tract. Consequently, maintaining an intestinal barrier homeostasis is crucial for controlling the state of chronic inflammation [3–5]. The gastrointestinal tract hosts a myriad of microorganisms that release pathogen-associated molecular patterns (PAMPs) and other metabolites [6], establishing the gut microbiota as a critical regulator of the immune system that also contributes to the metabolic health [7]. However, profound compositional and functional alterations in the gut microbiota together with a defective and impaired intestinal barrier accompanied by an increased intestinal permeability have been reported in obesity and its related metabolic disorders, including type 2 diabetes (T2D) [4, 8–10]. As a result of the intestinal barrier dysfunction, the altered microbiota or its metabolites translocate into the circulation instigating the low-grade inflammatory state [11]. In this regard, the presence of bacteria and commensal DNA have been detected in the blood and different AT depots in obesity and T2D [12, 13]. Metabolic endotoxemia also influences the lipid and glucose metabolism as well as vascular inflammation [7, 14]. In addition, research has also elucidated that the intestinal epithelium functions not only as a semi-permeable physical barrier but also exhibit immunological properties [15].

Mechanisms linking gut microbiota shifts and intestinal permeability with inflammation in obesity include the activation of innate immune cells by the capture of bacterial antigens. This process of bacterial translocation requires specific receptors including toll-like receptors (TLR) [16] and nod-like receptors (NLR) [17]. Opposite to the increased expression levels of TLRs and NLRs in innate immune cells, these receptors are generally expressed at low levels in enteroendocrine, goblet, Paneth and intestinal epithelial cells with a temporal and spatial regulation in response to diverse danger signals [18, 19]. The NLRP family is a subset of NLR characterized by an N-terminal pyrin domain and constituting important components of the inflammasomes, intracellular complexes that mediate innate immunity [20]. Diverse types of inflammasomes have been reported based on their main component, exhibiting different and complex roles depending on the tissue or the period they are activated [21]. Assembly of the NLRP3 inflammasome promotes the release of interleukin (IL)-1β and IL-18 by the activation of caspase-1 [20, 21]. Due to its potent inflammatory activity, IL-1 β contributes to the development of T2D [22]. Recently, our group has described that the blockade of the NLRP3 inflammasome reduces AT inflammation with significant fibrosis attenuation [23]. However, the role of the NLRP3 inflammasome in gut barrier disruption during intestinal inflammation in the context of obesity remains unsettled. While some studies have shown that *Nlrp3* knockout mice featured exacerbated colitis accompanied with decreased intestinal integrity and increased bacteria translocation from the gut to the systemic circulation [24, 25], other reports have demonstrated opposite results in disease severity [26]. Regarding the NLRP6 inflammasome, it is highly expressed in the small and large intestine and exhibits essential roles in the maintenance of intestinal homeostasis [27]. In this line, *Nlrp6*-deficient mice showed dysbiosis conferring increased susceptibility to colitis, colitis-associated colorectal cancer and metabolic syndrome development [11, 27–29]. Mechanistically, the activation of the NLRP6 inflammasome by metabolites derived from commensal bacteria maintains the homeostasis of the intestinal environment by controlling the release of mucus and antimicrobial peptides thereby regulating the microbiota composition [11, 30]. In addition, the NLRP6 inflammasome upregulation promotes the release of IL-18, that regulates intestinal inflammation as well as epithelial repair and defense against infections [11]. Interestingly, Seregin et al. [31] demonstrated that NLRP6 via IL-18 restricted the colonization of the mucolytic bacterium *Akkermansia muciniphila*, which is able to induce colitis in specific pathogen free and germ free-*Il10-*deficient mice. Oppositely, a well-documented role of *A. muciniphila* in maintaining the intestinal barrier integrity by modulating the host immune response and by regulating inflammation have been reported [32].

Current evidence shows that obesity-associated intestinal dysbiosis and permeability are crucial contributors of systemic chronic inflammation and end-organ dysfunction, also leading to metabolic diseases. In this context, the inflammasomes orchestrate either immunological tolerance or the induction of inflammatory responses to changes in gut microbiota. We hypothesized that intestinal permeability in obesity and T2D can dysregulate the host immune system through different mechanisms including the modulation of the inflammasome signaling contributing to metabolic inflammation. We therefore aimed to (i) analyze the potential changes in circulating concentrations of markers related to gut barrier dysfunction and its associated metabolic inflammation in a series of patients with obesity and T2D as well as the impact of weight loss; (ii) characterize the expression levels of the different components of the inflammasome, markers of integrity and inflammation of the intestinal epithelium in human jejunum samples in T2D, (iii) explore the effect of inflammation-related factors and bacteria-derived metabolites in the expression of inflammasomes in a non-cancerous cell line derived from the small intestine and (iv) to evaluate the effect of both, *A. muciniphila* and the *A. muciniphila*-conditioned medium on the intestinal integrity and inflammatory response in a small intestine cell line.

## Materials and methods

### Study population

Circulating levels of inflammation- and intestinal integrity-related factors were evaluated in a case–control study including 80 samples obtained from 35 males and 45 females from healthy normal weight (NW) volunteers (*n* = 20) or patients with obesity (OB) (*n* = 60) at the Clínica Universidad de Navarra. Body mass index (BMI) was calculated dividing weight (kilograms) by the square of the height (meters) and obesity was determined as a BMI ≥ 30 kg/m^2^. Body fat (BF) was assessed by air displacement plethysmography (Bod-Pod®, Life Measurements, COSMED, Italy). Following the guidelines of the American Diabetes Association [33], patients with OB were subclassified into two groups: with normoglycaemia (NG, *n* = 21) or with T2D (*n* = 39). Patients with T2D were newly diagnosed and were not under any type of pharmacotherapy that could modify their endogenous insulin levels. To our knowledge, patients were not on treatments that could potentially alter the integrity of the intestinal barrier [antibiotics, non-steroidal anti-inflammatory drugs (NSAIDs), oral contraceptive pills, chemotherapy drugs or proton pumps inhibitors]. Intraoperative jejunum biopsies were collected from patients with severe obesity undergoing a laparoscopic Roux-en-Y gastric bypass (RYGB) (NG, *n* = 5; T2D, *n* = 10) and were kept at − 80 °C. The collection of jejunum is clinically justified in this type of surgery since RYGB involves the creation of a small gastric pouch that is directly anastomosed to the jejunum.

To compare the impact of weight and fat loss achieved by RYGB or caloric restriction on circulating concentrations of intestinal dysfunction markers, blood samples from volunteers submitted to either RYGB (a subgroup of the previously described cohort, *n* = 20) or a conventional dietary treatment (*n* = 20) (both evaluated after 12 months) were used. Conventional dietary treatment consisted of a personalized diet prescribed by a physician in collaboration with a dietitian with planned regular follow-up visits to ensure a daily caloric deficit of 500–1000 kcal.

The protocol of the research was conformed to the guidelines of the Declaration of Helsinki and was approved by the Universidad de Navarra’s Ethical Committee (protocol 2020.054). All the participants signed the written informed consent.

### Analytical measurements

Plasma/serum samples were obtained after overnight fasting. Biochemical tests to determine the carbohydrate, lipid, hepatic and inflammatory profiles were accomplished as reported before [34]. Glucose was analyzed by an automated analyzer (Hitachi Modular P800, Roche, Basel, Switzerland). Insulin was measured by means of an enzyme-amplified chemiluminescence assay (IMMULITE®, Diagnostic Products Corp., Los Angeles, CA). Insulin resistance and sensitivity were calculated using the HOMA and QUICKI indices, respectively. Alanine aminotransferase (ALT), aspartate aminotransferase (AST), alkaline phosphatase (ALP) and γ-glutamyltransferase (γ-GT) were measured by enzymatic tests in an automated analyzer (Roche/Hitachi Modular P800). Leptin was measured by a double-antibody RIA method (Linco Research, Inc., St. Charles, MO, USA). Circulating levels of C–C motif chemokine ligand 5 (CCL5/RANTES) (R&D Systems, Minneapolis, MN, USA), endotoxin (Lonza Bioscience, Morrisville, NC, USA), flagellin (Cusabio, Houston, TX, USA), IL-1β (RayBiotech Life Inc., Peachtree Corners, GA, USA), IL-6 (RayBiotech Life Inc), IL-18 (RayBiotech Life Inc), IL-18-binding protein (IL-18BP) (R&D Systems), lactoferrin (Biovendor, Brno, Czech Republic), lipopolysaccharide-binding protein (LPB) (Hycult Biotech, Uden, The Netherlands), S100 calcium-binding protein A8 (S100A8/Calprotectin A) (R&D Systems) and zonulin (Inmunodiagnostik, Manchester, NH, USA) were measured by ELISA kits [35, 36]. For all the analysed molecules, the intra- and inter-assay coefficients of variation were < 12.0% and 15.0%, respectively.

### Analysis of gene expression levels

The isolation of total RNA and the synthesis of the first cDNA strand was performed as previously reported [23]. Briefly, RNA isolation and purification were performed using TRIzol^®^ Reagent (Invitrogen, Carlsbad, CA, USA) and RNeasy Mini Kit (Qiagen, Maryland, MD, USA), according to the manufacturer’s instructions. Samples were treated with DNase I (RNase Free DNase set, Qiagen). Constant amounts of 3 μg of total RNA were reverse transcribed using random hexamers (Roche) as primers and 300 units of M-MLV reverse transcriptase (Invitrogen). Gene expression levels of adiponectin (*ADIPOQ*), CD68 antigen (*CD68*), claudin 1 (*CLDN1*) *IL1B*, *IL18*, *IL33*, Kruppel-like factor 4 (*KLF4*), monocyte chemoattractant protein-1 (*CCL2*), mucin-2 (*MUC2*), lipocalin 2 (*NGAL/LCN2*), nucleotide-binding oligomerization domain, leucine-rich repeat and pyrin (*NLRP*)*-1, NLRP3, NLRP6,* nucleotide-binding oligomerization domain containing 2 (*NOD2*), occludin 1 (*OCLN1*), osteopontin (*SPP1*), *S100A8*, *S100A9*, six-transmembrane epithelial antigen of prostate 4 (*STEAP4*), tight junction protein 1 (*TJP1/ZO1*), toll-like receptor-4 (*TLR4*) were analyzed by Real-Time PCR (7300 Real Time PCR System, Applied Biosystem, Foster City, CA, USA). Primer Express 2.0 software (Applied Biosystems) was use do design primers and probes (Merck, Darmstadt, Germany) (Table S1), with the probes encompassing the ends of two exons to avoid possible genomic DNA amplification. The cDNA was amplified as previously described [23] using the TaqMan^®^ Universal PCR Master Mix (Applied Biosystems). The concentrations of primers and probes for gene amplification were 300 nM and 200 nM, respectively. The endogenous control gene *18S* rRNA (Applied Biosystems) was the loading control for Real-Time PCR experiments and relative quantification was calculated using the ΔΔCt formula.

### Western-blot

Total protein lysates from the jejunum (20 μg) were separated by Criterion™ TGX™ Precast Gels (Bio-Rad Laboratories, Inc., Hercules, CA, USA) and transferred to polyvinylidene difluoride (PVDF) membranes (Bio-Rad) [34]. Blots were blocked in Tris-buffered saline with Tween 20 (TBS-T) containing 5% BSA for 1 h at room temperature (RT), and then incubated overnight at 4 °C with rabbit monoclonal anti-NLRP3 (D2P5E Cell Signalling Technology Inc., Danvers, MA, USA) or polyclonal anti-NLRP6 (PA5-60466, Invitrogen, Paisley, UK). The antigen–antibody complexes were visualized using the goat anti-rabbit horseradish peroxidase (HRP)-conjugated antibodies (Amersham Biosciences, Buckinghamshire, UK) and the Pierce™ ECL Plus Western-blotting Substrate (Thermo Scientific, Rockford, IL, USA). The intensity of the bands was determined by densitometric analysis with the ChemiDoc™ MP imagining system and the Image Lab 4.0.1 software (Bio-Rad) and normalized with total protein values.

### Histological analysis

CD68, NLRP3 and NLRP6 immunodetection was performed in sections of formalin-fixed paraffin-embedded jejunum (4 µm) using the indirect peroxidase method as described before [34]. Jejunum sections from patients with obesity with and without T2D were incubated overnight at 4 °C with rabbit monoclonal anti-CD68 (ab213363, abcam, Cambridge, UK), monoclonal anti-NLRP3 or polyclonal anti-NLRP6 diluted 1:100 in Tris buffered saline (TBS, Merck). After washing, slides were incubated with anti-rabbit secondary antibodies conjugated with DAKO Real EnVision™ horseradish peroxidase (DakoCytomation, Glostrup, Denmark) for 1 h at RT. The peroxidase reaction was visualized using a 0.5 mg/mL diaminobenzidine/0.03% H_2_O_2_ solution diluted in 50 mmol/L Tris–HCl, pH 7.36, and Harris hematoxylin solution (Sigma) as counterstaining. Sections observed under a Zeiss Axiovert CFL light microscope (Zeiss, Göttingen, Germany) at 100X and 200X. A negative control slide was included in which the primary antibody was replaced by TBS to assess nonspecific staining. ImageJ analysis software (NIH, USA, https://imagej.net/ij/download.html) was used for quantitative evaluation. In addition, immunofluorescence staining was performed to provide a more specific localization of NLRP3, NLRP6 and CD68 in jejunum biopsies. To accomplish the immunofluorescence, after washing with TBS, slides were incubated with the goat anti-rabbit IgG secondary antibody Alexa Fluor^®^ 555 conjugate (abcam) (1:100 in TBS) during 30 min at RT. Alexa Fluor^®^ 555 conjugate was a generous gift from Dr. Marián Burrell and Dr. Marina Martín of the University of Navarra. Finally, sections were washed in TBS and mounted using DAPI (Invitrogen) as an aqueous mounting medium. To study the localization pattern of NLRP3, NLRP6 and CD68, an inverted microscope Nikon Eclipse-T300 was used. Images were recorded by the Digital sight DS-5MC camera with NIS-D software. Negative control slides without primary antibody were included to assess non-specific staining.

### Adipocyte and CCL-241 cell cultures

Stroma-vascular fraction cells were isolated from visceral AT from patients with OB and differentiated to adipocytes as described before [23]. The adipocyte conditioned media (ACM) was prepared by collecting the supernatant of differentiated adipocytes and further centrifuged and diluted (20% and 40%). ACM was used to evaluate the effects of the adipocyte secretome on the expression of inflammasome components in CCL-241, a non-cancerous small intestine cell line. CCL-241 cell line was obtained from the ATCC® (Middlesex, UK) and cultured at 37 °C in RPMI-1640 (Merck) supplemented with 10% fetal bovine serum and 30 ng/mL of the epidermal growth factor (Merck). Differentiated CCL-241 cells were serum starved for 24 h and then treated with increasing concentrations of TNF-α (1, 10 and 100 ng/mL; Merck), IL-1β (1, 10 and 100 ng/mL; R&D Systems), glucose (5, 10 and 25 nM; Merck), insulin (1, 10 and 100 ng/mL; Merck), rosiglitazone (10, 20 and 50 µM; Merck) and with taurine (70 mM, Merck) and histamine (25 mM, Merck), well-known activator and inhibitor of the NLRP6 inflammasome, respectively. Finally, CCL-241 cells were cultured in the presence of lipopolysaccharide (LPS) (1000 ng/mL) and parthenolide (PTL, a herbal NF-κB inhibitory compound that also inhibit the activity of multiple inflammasomes) (10 nM, Merck) for 4 h as well as with LPS for 3 h followed the addition of PTL for another 4 h.

### CCL-241 transfection with siNLRP6

CCL-241 cells were serum-starved for 2 h and then two pairs of small interfering RNAs (siRNAs) for blocking *NLRP6* expression (s46905 and s46907, Ambion, ThermoFisher Scientific, Waltham, MA, USA) were annealed and transfected into CCL-241 cells (200 pmol/L siRNA/2 × 10^5^ cells/well) using 40 nmol of Lipofectamine 2000 (Invitrogen, ThermoFisher). A scrambled siRNA was used as a negative control. The treatment with the *NLRP6*-s46905 and *NLRP6*-s46907 resulted in 72% and 61% average knockdown of the *NLRP6* mRNA, respectively (Fig. S1). Thus, the *NLRP6*-siRNA s46905 was chosen for *NLRP6* knocking-down studies.

### *Akkermansia munciniphila* culture and cells treatment

*Akkermansia muciniphila* (ATCC^®^ BAA-835™) was aseptically and anaerobically cultured in 6 mL tubes of brain heart infusion (BHI) broth (Becton Dickinson, Franklin Lakes, NJ, USA) at 37 °C for 7 days. Cultures were washed and concentrated in anaerobic phosphate buffered saline (PBS) (Merck) and heat-inactivated for 30 min at 70 °C. The bacteria-conditioned medium (BCM) was obtained by collecting the supernatant. The BCM was centrifuged and diluted at 40% in RPMI-1640 (Merck) (for the treatment of CCL-241 cells) and in DMEM/F-12 (for adipocyte treatment). CCL-241 cells and visceral adipocytes were serum-starved for 2 h and 24 h, respectively, and incubated with pasteurized *A. munciniphila* at a multiplicity of infection (MOI) of 100 as well as with the BCM (40%) for 4 h. The BHI medium (diluted at 40% in RPMI-1640 or in DMEM/F-12) was used as control medium. The conditioned media in both cell cultures were collected, centrifuged at 200 g for 10 min and stored at  − 80 °C. A commercially available ELISA kit (Mybiosource, San Diego, CA, USA) to assess the concentrations of mucin-2 in the media was used according to the manufacturer’s instructions.

### Animal model of diet-induced obesity

Four-week-old male Wistar rats (*n* = 40) were caged individually under controlled temperature, humidity, ventilation and light–dark cycles as previously described [37]. Animals were fed ad libitum either a chow normal diet (ND) (*n* = 10) (2014S, Harlan, Teklad Global Diets, Harlan Laboratories Inc., Barcelona, Spain) or a high-fat diet (HFD) (*n* = 30) (F3282, Bio-Serv, Frenchtown, NJ, USA) [38]. Rats were sacrificed and the small intestine (duodenum, jejunum and ileum) and blood samples were collected. The procedures followed the European Guidelines for the care and use of Laboratory Animals (directive 2010/63/EU) and were approved by the Ethical Committee for Animal Experimentation of the University of Navarra (026-19).

### Data and statistical analysis

The sample size was calculated using the G*Power 3.1.9.4 program (Franz Faul, University of Kiel, Germany) with preliminary data obtained in our own experience [23]. One-way ANOVA followed by Tukey’s post hoc tests was used to analyze differences in the circulating levels of inflammation- and intestinal integrity-related factors and one-way ANOVA followed by Dunnett’s post hoc tests to study differences in the in vitro experiments. Two-tailed unpaired Student’s* t* tests were applied to determine differences in the gene expression levels in jejunum samples. Correlations between two variables were computed by Pearson’s correlation coefficients. Calculations were performed with SPSS/Windows version 23 (Chicago, IL, USA) and graphs were created with GraphPad Prism version 8.3 (GraphPad Software, Inc., San Diego, CA, USA). A *P* value < 0.05 was considered statistically significant.

## Results

### Obesity and obesity-associated T2D drive an increase of intestinal damage markers

Clinical characteristics of the study population are summarized in Table 1. As expected, markers of adiposity were significantly higher (*P* < 0.001) in patients with OB compared to the volunteers with NW. In addition, OB was associated with adverse carbohydrate, lipid, inflammatory and hepatic profiles as well as systemic inflammation, being aggravated in patients with OB and T2D.

**Table 1:** Anthropometric and biochemical characteristics of subjects included in the study

	NW	OB-NG	OB-T2D
*n* (male, female)	20 (7, 13)	21 (7, 14)	39 (21, 18)
Age (years)	42 ± 5	40 ± 3	45 ± 2
BMI (kg/m^2^)	22.3 ± 0.8	43.4 ± 1.7***	44.3 ± 1.0***
Body fat (%)	22.1 ± 1.9	52.6 ± 1.5***	49.5 ± 1.2***
Waist circumference (cm)	76 ± 3	119 ± 3***	132 ± 2***,^††^
WHR	0.80 ± 0.02	0.88 ± 0.02***	1.00 ± 0.01***,^††^
WHtR	0.45 ± 0.01	0.72 ± 0.02***	0.78 ± 0.01***,^†^
Fasting glucose (mg/dL)	88 ± 4	90 ± 2	130 ± 8**,^††^
2 h OGTT glucose (mg/dL)	–	119 ± 3	196 ± 8***
Fasting insulin (µU/mL)	6.9 ± 1.3	16.3 ± 2.1	34.2 ± 1.7
2 h OGTT insulin (µU/mL)	–	116.5 ± 1.6	154 ± 1.5
HOMA	1.5 ± 0.4	3.7 ± 0.5***	8.6 ± 0.8***,^†††^
QUICKI	0.375 ± 0.016	0.325 ± 0.006***	0.291 ± 0.005***,^†††^
HbA1c	–	5.57 ± 0.09	7.50 ± 0.32***
Triglycerides (mg/dL)	65 ± 9	99 ± 8	149 ± 9***,^††^
Cholesterol (mg/dL)	168 ± 6	198 ± 8	184 ± 5
LDL-cholesterol (mg/dL)	95 ± 8	126 ± 7*	111 ± 4
HDL-cholesterol (mg/dL)	68 ± 5	53 ± 3*	44 ± 2***,^†^
Leptin (ng/mL)	8.1 ± 1.8	61.6 ± 6.3***	43.5 ± 6.1**
Leucocytes (× 10^6^)	6.72 ± 0.54	7.47 ± 0.48	7.84 ± 0.36
CRP (mg/L)	1.5 ± 0.5	9.7 ± 2.3*	8.8 ± 1.4*
Fibrinogen (mg/dL)	167 ± 34	385 ± 25**	423 ± 18***
von Willebrand factor (%)	38 ± 10	157 ± 39*	154 ± 10*
Homocysteine (µmol/L)	5.2 ± 0.4	9.1 ± 0.7	11.6 ± 1.1*
AST (U/L)	14 ± 1	22 ± 3	21 ± 2
ALT (U/L)	10 ± 4	29 ± 6	31 ± 3*
AST/ALT	1.88 ±0.27	0.84 ± 0.05**	0.76 ± 0.04**
γ-GT (U/L)	12 ± 2	28 ± 6	44 ± 9

Data are mean ± SEM. CRP concentrations were logarithmically transformed for statistical analysis. Differences between groups were analyzed by one-way ANOVA followed by Tukey’s post hoc tests or by unpaired two-tailed Student’s *t* tests, where appropriate

*ALT* alanine aminotransferase, *AST* aspartate aminotransferase, *BMI* body mass index, *CRP* C-reactive protein, γ*-GT* γ-glutamyltransferase, *HBA1c* glycated haemoglobin, *HOMA* homeostatic model assessment, *NG* normoglycemia, *NW* normal weight, *OB* obesity, *OGTT* oral glucose tolerance test, *QUICKI* quantitative insulin sensitivity check index, *T2D* type 2 diabetes, *WHR* waist-to-hip ratio, *WHtR* waist-to-height ratio

*P < 0.05, **P < 0.01 and ***P < 0.001 vs NW. ^†^*P* < 0.05, ^††^*P* < 0.01 and ^†††^*P* < 0.01 vs OB-NG

**Fig. 1:**
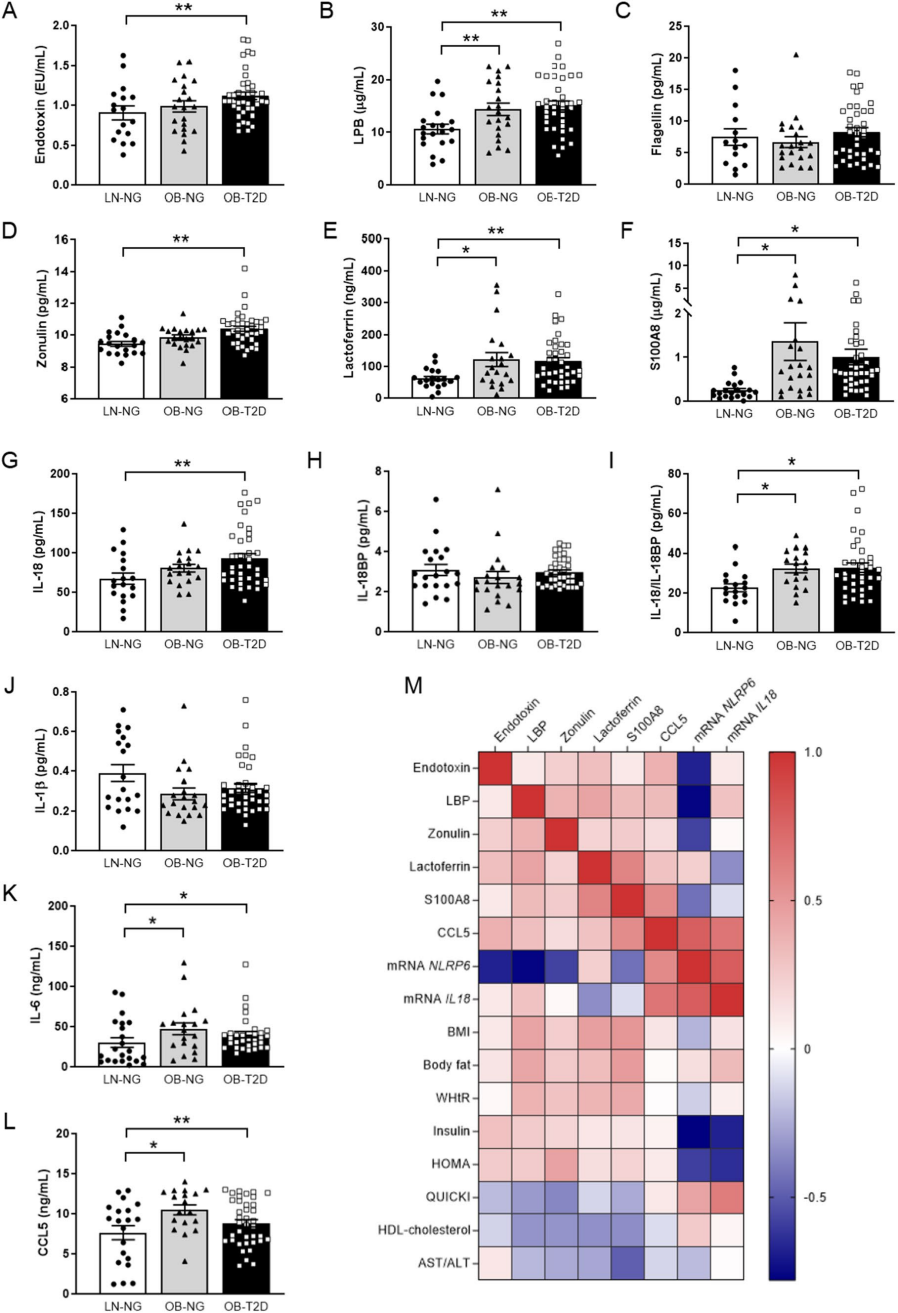
Effect of obesity and obesity-associated T2D on circulating levels of main markers related to intestinal dysfunction. Fasting plasma concentrations of **A** endotoxin, **B** lipopolysaccharide binding protein (LBP), **C** flagellin, **D** zonulin, **E** lactoferrin, **F** S100 calcium-binding protein A8/calprotectin A (S100A8), **G** interleukin-18 (IL-18), **H** interleukin-18 binding protein (IL-18BP), **I** ratio IL-18/IL-18BP, **J** interleukin-1β (IL-1β), **K** interleukin-6 (IL-6) and **L** C–C motif chemokine ligand 5 (CCL5/RANTES) in normal-weight (NW) volunteers (*n* = 17–20), patients with obesity with normoglycemia (OB-NG) (*n* = 21) and with obesity-associated type 2 diabetes (OB-T2D) (*n* = 39). **M** Heatmap of the associations between markers of intestinal dysfunction, gene expression levels of *NLRP6* and *IL18* in jejunum and anthropometric parameters, glucose profile as well as HDL-cholesterol and AST/ALT ratio. Bars represent the mean ± SEM. Differences between groups were analyzed by one-way ANOVA followed by Tukey’s tests. *P < 0.05 and **P < 0.01. *ALT* alanine aminotransferase, *AST* aspartate aminotransferase, *BMI* body mass index, *HOMA* homeostatic model assessment, *NLRP* nucleotide-binding oligomerization domain, leucine rich repeat and pyrin, *QUICKI* quantitative insulin sensitivity check index, *WHtR* waist-to-height ratio

Regarding intestinal dysfunction markers (Fig. 1A–F), both groups of patients with obesity showed higher circulating levels of LPB (*P* < 0.01), lactoferrin (*P* < 0.05 for OB-NG and *P* < 0.01 for OB-T2D) and S100A8 (*P* < 0.01). Elevated concentrations of endotoxin (*P* < 0.01) and zonulin (*P* < 0.01) were found in patients with obesity-associated T2D. No effect of gender was observed for all the analyzed molecules. Correlation analyses revealed a significant association between the circulating levels of the analyzed intestinal dysfunction factors (*P* < 0.01) and also with anthropometric determinations (*P* < 0.01) (Table S2, Fig. 1M). Importantly, we also detected that circulating levels of endotoxin, LBP, zonulin and S100A8 were associated with insulin resistance, due to the positive correlation with insulin levels and HOMA index and the negative association with QUICKI index (Table S2, Fig. 1M). Strong negative associations (*P* < 0.01) between HDL-cholesterol and LBP, zonulin, lactoferrin and S100A8 levels were also found, suggesting a potential role of these molecules in the regulation of lipid metabolism (Table S2, Fig. 1M). The AST/ALT ratio was negatively associated with zonulin (*P* = 0.044), lactoferrin (*P* = 0.037) and S100A8 (*P* < 0.001) levels (Table S2, Fig. 1M). Circulating levels of the intestinal inflammatory factors CCL5 and IL-6 as well as the IL-18/IL-18BP ratio were increased (*P* < 0.05) in patients with obesity with and without T2D (Fig. 1G–L) being also significantly associated with endotoxin levels.

To analyse the impact of therapeutic interventions aimed at achieving fat loss and metabolic improvement, the effect of conventional diet and RYGB on circulating concentrations of intestinal dysfunction markers was evaluated. After an average post-treatment period of 12 months, patients showed a significant decrease in all anthropometric measurements (*P* < 0.001) as well as a significant improvement in insulin resistance (*P* < 0.01) in patients submitted to bariatric surgery (Table S3). Noteworthy, a significant reduction in the circulating levels of endotoxin (*P* < 0.05), LBP (*P* < 0.01) and zonulin (*P* < 0.05) was observed after bariatric surgery (Fig. S2). Serum concentrations of LBP also decreased (*P* < 0.05) after dietary treatment.

### Decreased jejunal levels of NLRP6 in obesity-associated T2D

**Fig. 2:**
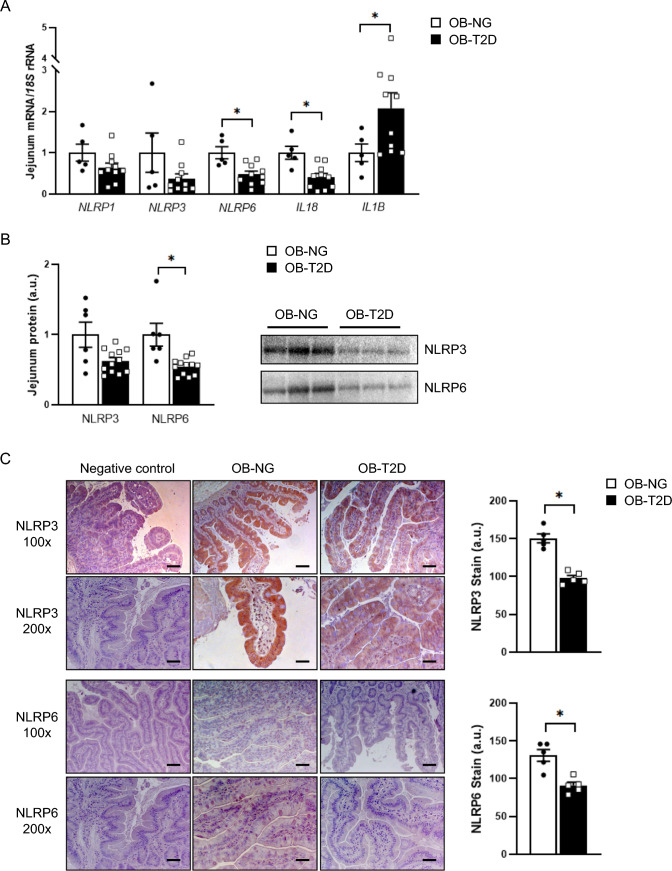
Impact of obesity-associated T2D on jejunum expression levels of the main inflammasome components. **A** Bar graphs show the gene expression levels of key inflammasome components (*NLRP1*, *NLRP3* and *NLRP6*) and its effectors (*IL1B* and *IL18*) in jejunum samples from patients with obesity with normoglycemia (OB-NG) (*n* = 5) and with obesity-associated type 2 diabetes (OB-T2D) (*n* = 10). **B** Protein expression levels of NLRP3 and NLRP6 in jejunum samples from patients with OB-NG (*n* = 5) and with OB-T2D (*n* = 10). **C** Representative immunostaining (*n* = 3) and quantification (*n* = 5 per group) for NLRP3 and NLRP6 in jejunum samples from patients with OB-NG and with OB-T2D [scale bar (100 × : 50 µm; 200 × : 25 µm)]. Values are the mean ± SEM. Differences between groups were analyzed by unpaired two-tailed Student’s *t* test. *P < 0.05. *NLRP*, nucleotide-binding oligomerization domain, leucine rich repeat and pyrin; *IL*, interleukin

To verify whether obesity-associated T2D is involved in the regulation of the inflammasome in the small intestine, gene expression levels of the main components of the inflammasome and its major effectors were determined in human jejunum samples. Results showed decreased gene expression levels of *NLRP6* (*P* < 0.05) and *IL18* (*P* < 0.05) together with an upregulation of *IL1B* (*P* < 0.05) in patients with T2D (Fig. 2A). Although mRNA levels of *NLRP1* and *NLRP3* were decreased in T2D, differences were not statistically significant. Importantly, mRNA levels of *NLRP6* were highly correlated with the expression of its main effector, *IL18* (*r* = 0.77; *P* = 0.002) (Table S2, Fig. 1M). Gene expression levels of *NLRP6* in the jejunum were negatively associated with circulating levels of endotoxin (*r* =  *− *0.68; *P* = 0.044) and LBP (*r* =  *− *0.77; *P* = 0.015). (Table S2, Fig. 1M), reinforcing the role of NLRP6 in the maintenance of the intestinal barrier integrity. We also found a negative association between the expression of *NLRP6* and *IL18* with insulin levels (*P* = 0.003 and *P* = 0.011, respectively) and HOMA index (*P* = 0.046 and *P* = 0.018, respectively). In addition, a positive correlation between *IL18* mRNA levels and QUICKI index was found (*P* = 0.021) (Table S2, Fig. 1M). The expression of *NLRP6* and *IL18* was also associated with circulating levels of CCL5 (*P* = 0.014 and *P* = 0.033, respectively) (Table S2, Fig. 1M). Decreased expression of NLRP6 in the jejunum from patients with T2D was corroborated at the protein level by Western-blot (Fig. 2B) and by immunohistochemistry (Fig. 2C). Remarkably, a different pattern of staining was observed between NLRP3 and NLRP6, with NLRP3 being primarily localized in the intestinal epithelial cells and NLRP6 being expressed in goblet cells (Fig. 2C). To gain further insight, the presence of NLRP3 and NLRP6 in sections of human jejunum was confirmed by immunofluorescence (Fig. S3A and S3B). OB-NG patients were immunopositive for NLRP3 and NLRP6. Although NLRP3 and NLRP6 levels were readily evident in the epithelial cells, an increased immunostaining was detected in the apical region of cells. No severe intestinal histological damage was observed in the jejunum from patients with obesity and with obesity-associated T2D. However, a higher number of inflammatory infiltrating cells together with a slight mucosal sloughing were observed in patients with T2D.

We next confirmed reduced levels of mRNA levels of *Nlrp1*, *Nlrp3* and *Nlrp6* in the duodenum, jejunum and ileum of HFD-fed rats (Fig. S4). As expected, HFD feeding increased adiposity (ND-fed rats 558 ± 24 g vs HFD-fed rats 630 ± 15 g, *P* < 0.05) and induced insulin resistance as evidenced by higher insulin levels (ND-fed rats 5.4 ± 0.5 ng/mL vs HFD-fed rats 5.8 ± 1.1 ng/mL, *P* < 0.05) and HOMA index (ND-fed rats 1.3 ± 0.1 vs HFD-fed rats 1.5 ± 0.3 *P* < 0.05) in the animal model. Similar to human samples, we confirmed decreased (*P* < 0.05) mRNA levels of *Nlrp6* in the jejunum obtained from rats with DIO, with also a downregulation (*P* < 0.05) of *Nlrp1* and *Nlrp3* (Fig. S4B). In the duodenum and ileum, HFD-fed rats also exhibited decreased (*P* < 0.05) levels of *Nlrp6* (Figs. S4A, S4C).

### Dysregulation of inflammatory and intestinal integrity markers in jejunum in T2D

**Fig. 3:**
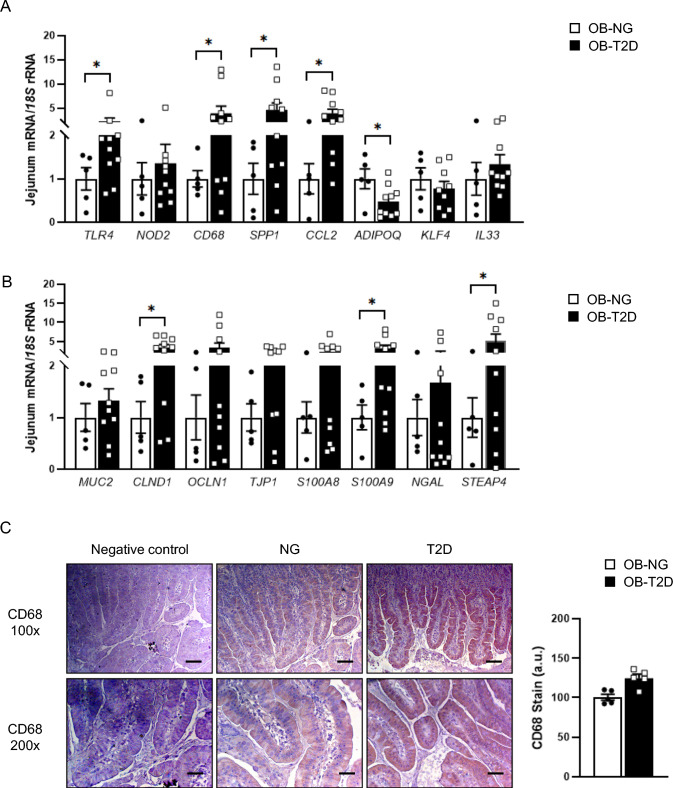
Bar graphs show the mRNA levels of **A** key intestinal inflammation-related genes and **B** markers associated with the integrity of the epithelial intestinal barrier in jejunum samples from patients with obesity with normoglycemia (OB-NG) (*n* = 5) and with obesity-associated type 2 diabetes (OB-T2D) (*n* = 10). **C** Representative immunostaining (*n* = 3) and quantification (*n* = 5 per group) for CD68 in jejunum samples from patients with OB-NG and with OB-T2D [scale bar (100 × : 50 µm; 200 × : 25 µm)]. Values are the mean ± SEM. Differences between groups were analyzed by unpaired two-tailed Student’s *t* test. *P < 0.05. *ADIPOQ* adiponectin, *CCL2* monocyte chemoattractant protein-1, *CLDN1* claudin 1, *IL* interleukin, *KLF4* Kruppel like factor 4, *MUC2* mucin 2, *NGAL* lipocalin 2, *NOD2* nucleotide binding oligomerization domain containing 2, *OCLN* occluding, *S100A9* S100 calcium-binding A9, *SPP1* osteopontin, *STEAP4* STEAP4 metalloreductase, *TLR4* toll-like receptor-4, *TJP1* tight junction protein-1

Since mounting evidence relates intestinal inflammation with the occurrence and progression of T2D, we analyzed the expression of inflammation-related factors in the jejunum from patients with obesity with or without T2D (Fig. 3). An upregulation of *TLR4* (*P* < 0.05), *CD68* (*P* < 0.05), *SPP1* (*P* < 0.05) and *CCL2* (*P* < 0.05) together with a downregulation (*P* < 0.05) of *ADIPOQ* levels in the jejunum from patients with obesity-associated T2D were found (Fig. 3A). Increased expression levels of CD68 in patients with T2D also were confirmed by immunohistochemistry (Fig. 3C) and immunofluorescence (Fig. S3C), with a higher number of inflammatory cells being detected in patients with T2D. In addition, mRNA levels of *STEAP4*, a metalloreductase involved in cellular copper and iron uptake in response to chronic inflammation and therefore, in maintaining gut homeostasis [39], was increased (*P* < 0.05) in the jejunum from patients with T2D. Gene expression levels of the calprotectin subunit *S100A9* were also higher (*P* < 0.05) in T2D. No differences were detected in the expression of either *MUC2* or the junction proteins *OCLN1* and *TJP1* but, unexpectedly, increased expression (*P* < 0.05) of *CLDN1* was found in the jejunum from T2D volunteers (Fig. 3B). Circulating zonulin levels were associated with the expression of *TLR4* (*r* = 0.82; *P* < 0.001), *CLDN1* (*r* = 0.69; *P* = 0.013) and *OCLN1* (*r* = 0.67; *P* = 0.017).

### NLRP6 is regulated by glucose-related metabolic factors as well as by taurine and parthenolide in small intestine cells

**Fig. 4:**
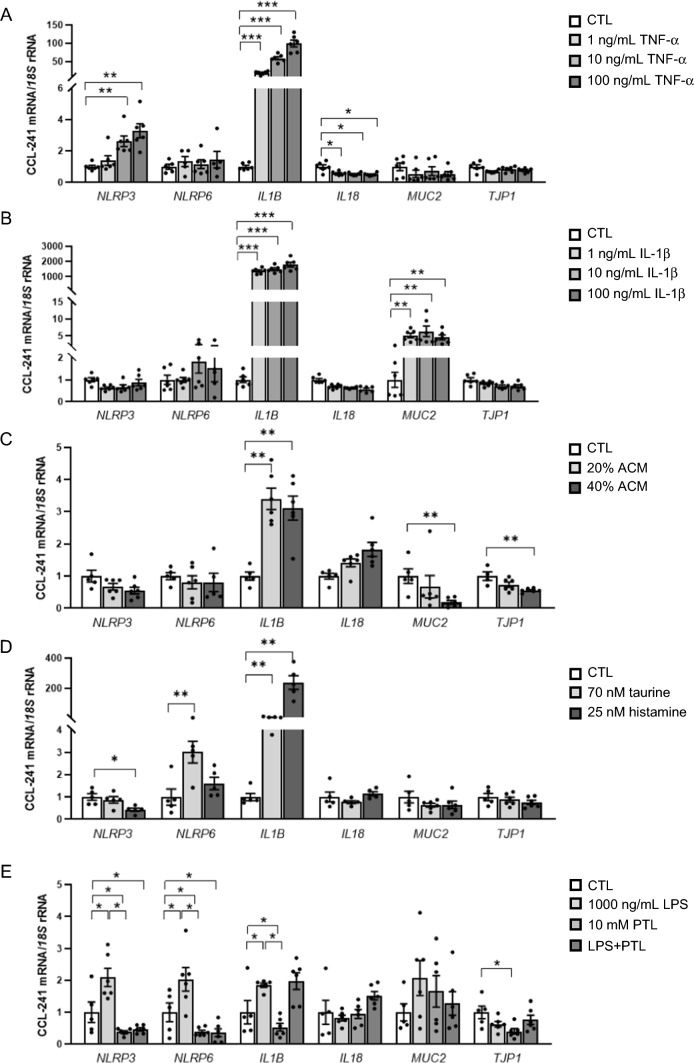
Gene expression levels of the inflammasome components (*NLRP3* and *NLRP6*), their main effectors (*IL1B* and *IL18*) and the intestinal integrity genes (*MUC2* and *TJP1*) in human enterocytes CCL-241 cells treated with different concentrations of **A** tumor necrosis factor (TNF)-α, **B** interleukin (IL)-1β, **C** adipocyte conditioned media (ACM) and **D** taurine and histamine during 24 h (*n* = 6 per group). **E** Effect of lipopolysaccharide (LPS), parthenolide (PTL) and LPS for 3 h followed by PTL for another 4 h in the regulation of inflammasome components and inflammation and intestinal integrity genes in CCL-241 cells. Values are the mean ± SEM (*n* = 6 per group). Differences between groups were analyzed by one-way ANOVA followed by Dunnett’s tests. *P < 0.05, **P < 0.01 and ***P < 0.001 vs unstimulated cells. *NLRP* nucleotide-binding oligomerization domain, leucine rich repeat and pyrin, *IL* interleukin, *MUC2* mucin 2, *TJP1* tight junction protein-1

We recreated an aspect of the intestinal inflammatory pathophysiology of the intestinal epithelium stimulating human CCL-241 enterocytes with the pro-inflammatory factors TNF-α and IL-1β as well as with the ACM obtained from patients with obesity, studying also the potential crosstalk between adipocytes and intestinal cells. As shown in Fig. 4A, TNF-α (*P* < 0.01) significantly increased the mRNA levels of *NLRP3* without changes in *NLRP6* expression. After TNF-α treatment, we also detected a strong increase (*P* < 0.0001) in the expression of *IL1B* together with a decrease (*P* < 0.05) in the mRNA levels of *IL18* (Fig. 4A). IL-1β significantly upregulated (*P* < 0.0001) its own expression as well as *MUC2* mRNA (*P* < 0.01) (Fig. 4B). After the treatment with the ACM, a slight decrease in the *NLRP3* and *NLRP6* inflammasome was observed but differences were not statistically significant (Fig. 4C). However, *MUC2* and *TJP1* expression levels were significantly downregulated (Fig. 4C). CCL-241 cells showed a significant increase (*P* < 0.01) in the mRNA levels of *NLRP6* in response to glucose and oppositely, after the stimulation with insulin, gene expression levels of *NLRP6* decreased (*P* < 0.05) (Fig. S5A, S5B). We also found that after the treatment with glucose and insulin, transcript levels of *IL1B* increased (*P* < 0.01 and *P* < 0.05, respectively) and the mRNA of *MUC2* was downregulated (*P* < 0.05 and *P* < 0.01, respectively). In addition, the stimulation of the human intestinal cell line with rosiglitazone significantly increased (*P* < 0.01) the expression of *NLRP6* (Fig. S5C).

In addition, we stimulated CCL-241 cells with an activator (taurine) and specific inhibitors (histamine and PTL) of the NLRPs. After the treatment with taurine, we found an increase (*P* < 0.05) in the expression of *NLRP6* with also a strong upregulation (*P* < 0.001) of *IL1B* (Fig. 4D). No effect of histamine was found in the regulation of *NLRP6* (Fig. 4D). Lastly, the treatment with PTL significantly inhibited (*P* < 0.05) the expression of *NLRP3* and *NLRP6* in the intestinal cell line and blocked LPS effects (Fig. 4E). mRNA levels of *IL1B* increased (*P* < 0.05) after LPS treatment and decreased (*P* < 0.05) with PTL but no differences were found in cells previously stimulated with LPS (Fig. 4E). *TJP1* expression levels were decreased (*P* < 0.05) after PTL treatment (Fig. 4E).

### *NLRP6* knockdown resulted in decreased expression and release of MUC2

**Fig. 5:**
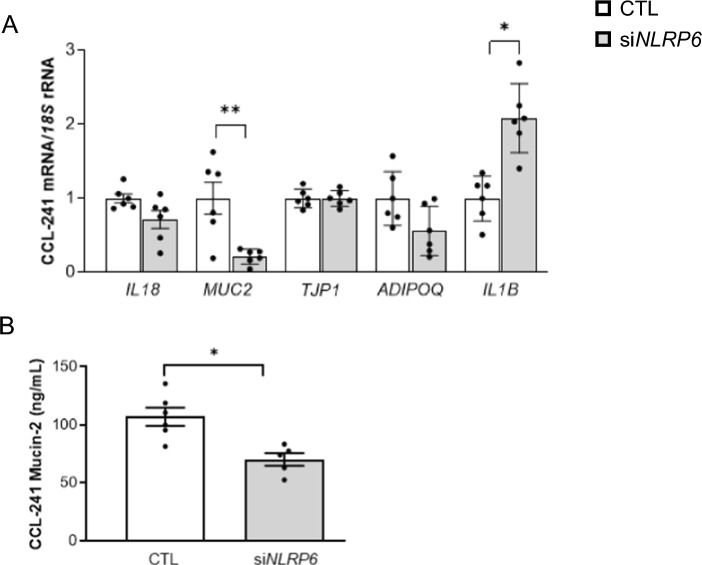
Effect of blocking *NLRP6* expression in **A** the gene expression levels of interleukin-18 (*IL18*), mucin 2 (*MUC2*), tight junction protein 1 (*TJP1*), adiponectin (*ADIPOQ*) and interleulin-1β (*IL1B*) and **B** the release of mucin-2 into the culture media. CCL-241 cells were transfected with or without 200 pmol/L *NLRP6* siRNA/2 × 10^5^ cells/well for 24 h. Values are the mean ± SEM (*n* = 6 per group). Differences between groups were analyzed by unpaired Student’s *t*-test. *P < 0.05 and **P < 0.01

Since gene expression levels of *NLRP6* were downregulated in the jejunum from patients with T2D, we reduced the constitutive expression levels of *NLRP6* in CCL-241 cells using a specific siRNA to get insight into its mechanism of action (Fig. 5). Although no differences regarding the expression level of *IL18* were found, a significant decrease (*P* < 0.01) in the expression of *MUC2* was observed (Fig. 5A). No differences were found in the mRNA levels of *ADIPOQ* and *TJP1*, while the expression of *IL1B* was significantly increased (*P* < 0.05). Importantly, we measured the secretion levels of mucin-2 into the culture medium finding a significant reduction after si*NLRP6* treatment (Fig. 5B).

### *Akkermansia muciniphila* increased the release of MUC2

**Fig. 6:**
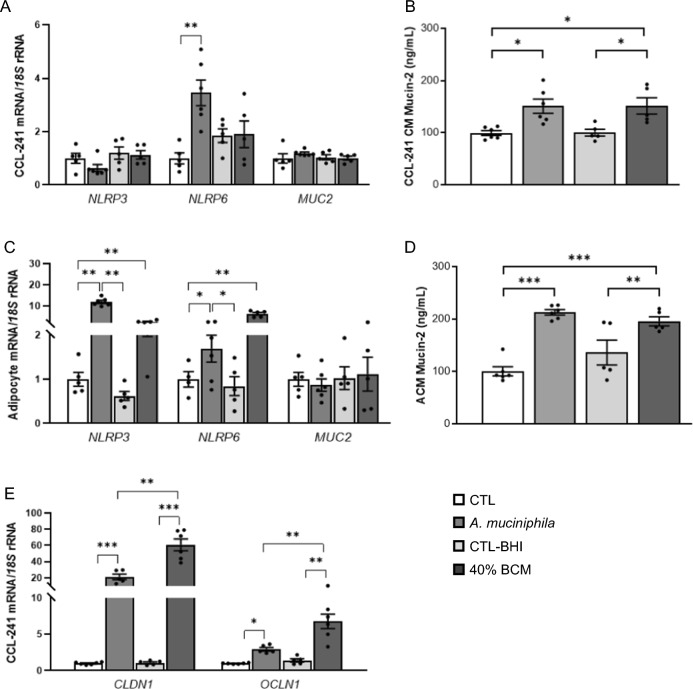
Gene expression levels of *NLRP3*, *NLRP6* and *MUC2* in **A** CCL-241 cells and **C** visceral adipocytes after the incubation with heat-inactivated *Akkermansia muciniphila* and with the bacteria-conditioned medium (BCM) (40%) for 24 h. Mucin-2 concentrations in the culture media of **B** CCL-241 cells and **D** visceral adipocytes incubated in the presence of heat-inactivated *A. muciniphila* and with the bacteria-conditioned medium (BCM) (40%) for 24 h. **E** Gene expression levels of *CLDN1 and OCLN* in CCL-241 cells after the incubation with heat-inactivated *Akkermansia muciniphila* and with the bacteria-conditioned medium (BCM) (40%) for 24 h. Values are the mean ± SEM (*n* = 6 per group). Differences between groups were analyzed by one-way ANOVA followed by Tukey’s tests. *P < 0.05, **P < 0.01 and ***P < 0.001. *BHI* brain heart infusion, *CLDN1* claudin 1, *MUC2* mucin 2, *NLRP* nucleotide-binding oligomerization domain, leucine rich repeat and pyrin, *OCLN* occludin

*Akkermansia muciniphila* has been associated with improvements in local and systemic inflammation as well as in intestinal barrier integrity [40, 41]. Therefore, both CCL-241 cells and adipocytes were treated with the heat-inactivated bacteria and with its conditioned media (Fig. 6). An increased (*P* < 0.01) gene expression level of *NLRP6* after the incubation of CCL-241 cells with heat-inactivated *A. muciniphila* was observed, whereas no differences in *NLRP3* or *MUC2* expression levels were detected (Fig. 6A). However, higher release of mucin-2 into the culture media after the treatment with both the bacteria and the BCM was found (Fig. 6B). Similar results were obtained in visceral adipocytes with upregulated expression of *NLRP3* and *NLRP6* after the treatments and increased levels of mucin-2 into the culture media (Fig. 6C and D). After the incubation of CCL-241 cells with both the heat-inactivated *A. muciniphila* and the BCM, a strong upregulation (*P* < 0.01) in the gene expression levels of *CLDN1* and *OCLN1*, two key molecules in the maintenance of the intestinal integrity, was observed (Fig. 6E).

## Discussion

Obesity and T2D are well-recognized conditions involved in the dysregulation of gut inflammation, intestinal epithelial cells integrity and adhesion and, therefore, intestinal barrier function, favoring the translocation of bacteria or harmful exogenous factors into circulation and constituting an important hit of metabolic inflammation [8, 42]. The main findings of our study are as follows: (i) obesity and T2D increased circulating levels of markers of intestinal integrity damage and inflammation being associated with insulin resistance and lipid metabolism, (ii) expression levels of *NLRP6* and *IL18* were decreased in the jejunum from patients with obesity and T2D together with an upregulation of inflammatory markers and (iii) we further revealed that NLRP6 regulation is likely context-dependent, with taurine increasing and parthenolide decreasing its expression levels in human CCL-241 enterocytes. Additionally, we also demonstrated decreased expression levels of *Nlrp6* in the small intestinal tract (duodenum, jejunum and ileum) from rats with DIO.

The determination of circulating bacteria-related components (such as LPS or flagellin) or direct intestinal barrier damage markers (zonulin, calprotectin or FABPs) constitutes an indirect measurement of increased intestinal permeability. Different studies have also corroborated the association between dysregulated intestinal permeability, increased visceral obesity and insulin resistance [9, 10, 43]. In this sense, we found higher levels of the bacterial component LPS in patients with obesity-associated T2D, with LPB, that recognizes and binds LPS, being also increased in obesity with and without T2D. Results from large cohorts have described augmented levels of LPS and LBP in subjects with metabolic syndrome or T2D [44–46], proposing their role as triggering factors of the early development of metabolic diseases. Moreover, an influx of bacteria-derived LPS into the systemic circulation has been demonstrated in mice fed a HFD [47]. In addition to LPS, zonulin is considered a biomarker of impaired gut barrier function being associated with chronic inflammation, insulin resistance and bacterial leakage due to its function as intercellular tight junctions regulator [48, 49]. In this regard, patients with obesity and T2D included in our study exhibited higher serum levels of zonulin. Other factors including calprotectin, a sensitive marker for mucosal inflammation of the intestine [50] or lactoferrin, a first-line defense protein for protection against microbial infections [51] were increased in our patients with obesity and T2D. Reportedly, pro-inflammatory chemokines exhibit important roles in the development of intestinal diseases even in colon carcinogenesis [52, 53]. An aberrant microbiota has been shown to induce the epithelial expression of CCL5, promoting a spontaneous and exaggerated autoinflammatory response [28]. We found increased levels of CCL5 in patients with obesity and obesity-associated T2D, with also higher levels of IL-6 and the ratio IL-18/IL-18BP. Taken together, these results suggest that the altered intestinal barrier demonstrated by the increased levels of LPS and key intestinal damage markers (LBP, zonulin, calprotectin, lactoferrin) in patients with a compromised metabolic state may cumulatively worsen their condition due to the greater exposure to endotoxins and pro-inflammatory factors. In accordance with previous reports [3, 10, 12, 47], the positive association found between the endotoxin LPS, LBP, zonulin and S100A8 with fasting insulin and the HOMA index as well as the negative correlation with the QUICKI underline the link between intestinal permeability and insulin resistance and, consequently, the development of T2D. Bariatric surgery directly alters the structure of the gastrointestinal tract from patients with obesity affecting the gut microbiome and the intestinal immune system [54]. In this sense, a reduction in serum concentrations of intestinal damage markers has been reported after bariatric surgery [55, 56]. The decrease in the circulating levels of endotoxin, LBP and zonulin found in our patients after RYGB suggests an improvement of the impaired intestinal permeability probably related to the alleviation of the comorbid conditions.

The opposite functions of the inflammasomes in the development of intestinal diseases are related with their roles in the protection/repair or damage of the intestinal mucosa, being the consequence of their opposed functions in different contexts and cellular types [24–26, 57]. While the proposed function of inflammasomes in intestinal epithelial cells consists in the regulation of the secretion of IL-18 by promoting the regeneration of the epithelial barrier, in hematopoietic cells, their activation may have a proinflammatory effect [57]. Moreover, depending on the level of the intestinal tissue damage, a shift in the balance between protective and detrimental effects has been proposed. Specifically, NLRP6, highly expressed in the intestine, executes essential roles for intestinal mucosal self-renewal and proliferation also protecting the host against bacterial and viral infection [58]. Elinav et al. [28] elegantly proposed that *Nlrp6* deficiency prompts an impairment of the intestinal barrier function mainly due to changes in the microbiota partly regulated by the secretion of IL-18. Moreover, *Il18* knockout mice are susceptible to the development of hyperphagia, obesity, and insulin resistance [59]. We found decreased levels of NLRP6 and its main effector IL-18 in the jejunum from patients with T2D, suggesting a defective sensor system to detect PAMPS and DAMPS that drives to a damaged epithelial barrier that increases intestinal permeability and allows leakage of bacteria or bacterial products. In addition, the expression levels of *NLRP6* and *IL18* were highly associated between them and also with insulin resistance, strengthening their involvement in glucose metabolism. *NLRP6* expression in adipose tissue and circulating levels of IL-18 were significantly upregulated in patients with NASH and portal fibrosis compared with patients without portal fibrosis [23, 60]. However, similar to NLRP6, the precise contributions of IL-18 to intestinal homeostasis and inflammation still remain controversial and unresolved. On one hand, complete loss of IL-18 predisposes mice to increased intestinal epithelial damage [28, 61] and on the other hand, IL-18 is a potent pro-inflammatory cytokine able to induce inflammation-related mediators [62]. Supporting the notion that CCL5 is upregulated in response to the altered microbiota in *Nlrp6*^−/−^ mice [28], we found an association between CCL5 circulating levels and gene expression levels of *NLRP6* and *IL18*. The involvement of NLRP6 in epithelial integrity has been linked to the regulation of goblet cell function by controlling mucus secretion [30] and interestingly, we found that NLRP6 was mainly localized in goblet cells, whereas NLRP3 was located in the epithelial cells. Reportedly, *Nlrp6*-deficient mice exhibited a defective mucus layer accompanied with the subsequent failure to remove pathogens and, thus, increasing the susceptibility to infections [30]. In line with the results found in human samples, a downregulation of *Nlrp6* in the jejunum from rats with DIO was found, which is in agreement with previous results showing that the reduced gene expression levels of intestinal *Nlrp6* in obesity has been efficiently reversed by RYGB but not by caloric restriction [63].

Parallel to obesity and insulin resistance is the low-grade inflammatory status [64]. Recent evidence supports the concept that alterations in the microbiota due to obesity promote early inflammatory changes in the small intestine that also gives susceptibility to insulin resistance [65]. Mice deficient in *Nlrp6* are also more prone for intestinal inflammation and features of the metabolic syndrome mainly due to dysbiosis [28, 29, 66]. We showed that gene expression levels of crucial inflammatory mediators including *CCL2*, *CD68*, *IL1B*, *SPP1* and *TLR4* were increased in the jejunum from patients with T2D with a downregulation of the anti-inflammatory marker *ADIPOQ*. Homeostasis of iron metabolism is of great importance for intestinal inflammation and an overexpression of the metalloreductase *STEAP4* has been associated with aggravated inflammatory bowel disease [39]. Accordingly, we detected higher levels of *STEAP4* in the jejunum in T2D. Additionally, impaired expression and distribution of tight junctions in the epithelium of jejunum has been described as an early event in prediabetes development, occurring even without endotoxemia [67]. We did not find changes in the expression of *OCLN1* and *TJP1* but upregulated levels of *CLDN1* were observed in patients with T2D, probably as a compensatory response to prevent intestinal damage. Another essential target to avoid intestinal diseases is the mucus layer, with goblet cells being the specialized intestinal cells involved in the production and release of mucins, mostly MUC2 [30, 68]. However, no differences were observed in *MUC2* levels in patients with T2D.

Host- and microbiota-derived metabolites participate in the control of NLRP6 inflammasome signaling [66]. Indeed, potential metabolites that activate the inflammasomes included the bile acid derivate taurine, carbohydrates, and long-chain fatty acids, whereas histamine and spermine are considered robust inhibitors of IL-18 release [66]. Neither the pro-inflammatory factors TNF-α and IL-1β nor the secretome from patients with obesity (that is enriched in inflammatory mediators) exhibited an effect on the modulation of the NLRP6 inflammasome in the human enterocyte cell line CCL-241. Importantly, the ACM obtained from patients with obesity downregulated the expression levels of *TJP1* and *MUC2*, suggesting a crosstalk between adipocytes and intestinal cells in which the pro-inflammatory profile of adipocytes may impair the integrity of intestinal barrier. The increased expression of *NLRP3* and its direct mediator *IL1B* together with the downregulation of *IL18* mediated by TNF-α point to the effect of this cytokine in promoting intestinal inflammation. Peroxisome proliferator-activated receptor (PPAR)-γ is involved in intestinal homeostasis by preventing inflammation [69]. Reportedly, the administration of rosiglitazone, a PPAR-γ agonist commonly used as an insulin-sensitizer in the management and treatment of T2D, to rodents exerted protective effects in chronic experimental colitis [70]. According to our results, Caco2 cells showed a significant increase in *NLRP6* mRNA that was dose dependent in response to rosiglitazone [71] suggesting that protective effects of PPAR-γ may be mediated by increasing the expression of *NLRP6*. Taurine, previously proposed for increased NLRP6 activity [66], significantly enhanced the expression levels of *NLRP6*. The amino acid histamine had no effect on *NLRP6* mRNA levels in CCL-241 cell line. Parthenolide, a strong inflammasome inhibitor independent of its inhibitory effect on the NF-κB pathway [72] induced a decrease in the expression levels of *NLRP3* and *NLRP6* in CCL-241 cells even being stimulated with LPS.

Although *A. muciniphila* has been associated with improvements in local and systemic inflammation, *Nlrp6*-deficient mice are more susceptible to colitis due to an increase in *A. muciniphila* in the gut. We found an upregulation of *NLRP6* after the incubation of CCL-241 cells with heat-inactivated *A. muciniphila* whereas no differences in *NLRP3* or *MUC2* expression levels were detected. However, a higher release of mucin-2 into the culture media was found after the treatment with both the bacteria and the BCM. These results confirm the complex regulatory pathways of the inflammasomes and highlight the importance of the relative contribution of each metabolite to determine the overall activation of the inflammasomes and the production of their downstream targets.

*Nlrp6* knockout mice exhibited a defect in the exocytosis of mucin granules due to reduced autophagy and hyperplasia of goblet cells, resulting in a thin mucus layer and higher susceptibility to infections [30, 73]. In this sense, we observed a decrease in the expression and release of MUC2 after the treatment with si*NLRP6*, strengthening the role of NLRP6 in mucin release and, therefore, in the maintenance of gut homeostasis.

The important regional differences found along the proximal–distal axis in the gut regarding cellular composition and gene expression levels, highlight the importance of regional selection when studying the gut [74]. In this sense, a specific part of the jejunum has been collected in the patients included in our study. However, to further clarify the exact intestinal region and cellular type responsible for the activation of the inflammasomes will help to understand how inflammation affects the intestinal epithelium and will be a guidance for future precision medicine approaches. In addition, transepithelial electrical resistance functional assays to measure the integrity of tight junction dynamics as well as to evaluate the impact of inflammatory factors and/or different microbes on the intestinal permeability will be highly relevant and crucial in developing strategies to solve epithelial barrier dysfunctions.

## Conclusions

**Fig. 7:**
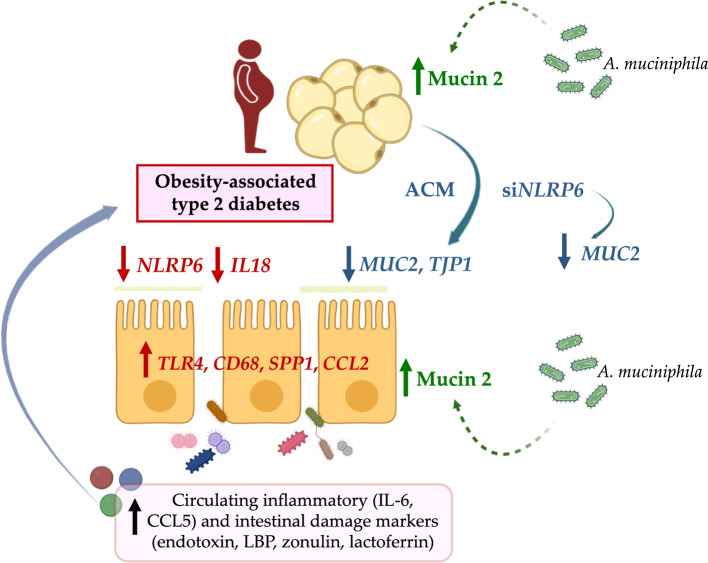
The downregulated expression of *NLRP6* and *IL18* and increased levels of pro-inflammatory factors (*TLR4*, *CCL2*, *SPP,* and *CD68*) in the jejunum from patients with obesity-associated T2D suggest a defective inflammasome sensing, driving to an impaired epithelial intestinal barrier, also evidenced by the increased circulating levels of intestinal damage markers (endotoxin, LBP, zonulin, lactoferrin, S100A8) and inflammatory factors (IL-6, CCL5) favoring the development of multiple obesity-associated comorbidities. In addition the secretome of adipocytes from patients with obesity-associated T2D decreased the expression of *MUC2* and *TJP1* in intestinal cells, strengthening the role of adipocytes in the maintenance of the intestinal barrier homeostasis and the crosstalk between these types of cells. *CCL2*, monocyte chemoattractant protein-1; CCL5, C–C motif chemokine ligand 5; *NLRP* nucleotide-binding oligomerization domain, leucine rich repeat and pyrin, *IL* interleukin, *LBP* lipopolysaccharide binding protein, *MUC2* mucin 2, *S100A8* S100 calcium-binding A8, *SPP1* osteopontin, *TJP1* tight junction protein-1, *TLR4* toll-like receptor-4

Clinical and translational studies have provided evidence that the dysregulation of the inflammasomes in the gastrointestinal tract may play an important role in obesity and metabolic disorders. Collectively, the increased circulating levels of intestinal damage markers together with the downregulated expression of *NLRP6* and *IL18* and increased levels of pro-inflammatory factors in the jejunum from patients with obesity-associated T2D suggest a defective inflammasome sensing, driving to an impaired epithelial intestinal barrier and uncontrolled inflammation that may regulate the progression of multiple obesity-associated comorbidities (Fig. 7). Further research is warranted to understand the cell-, tissue- and time-specific functions of NLRPs and to apply our knowledge of inflammasome biology to diminish the inflammation-induced tissue injury in different conditions including obesity, inflammatory bowel disease or even inflammation-associated cancers. Since conflicting results of NLRP6 in the control of the intestinal integrity, in the responses against microbial pathogens and in the inner colonic inner mucus layer formation have been proposed, studies to understand the dichotomic roles in inflammation mediated by NLRP6 are also essential [75, 76]. Our data suggest that to increase the reduced intestinal expression of *NLRP6* in patients with obesity-associated T2D may be a potential therapeutic intervention.

### Supplementary Information

Below is the link to the electronic supplementary material.

Supplementary file1 (DOCX 565 KB)

## Data Availability

The datasets used and/or analysed during the current study are available from the corresponding author on reasonable request.

## Abbreviations

ACM: Adipocyte conditioned media
ADIPOQ: Adiponectin
AT: Adipose tissue
BMI: Body mass index
CCL2: Monocyte chemoattractant protein-1
CD68: CD68 antigen
CLDN1: Claudin 1
CRP: C-reactive protein
DIO: Diet-induced obesity
IL: Interleukin
KLF4: Kruppel-like factor 4
LBP: Lipopolysaccharide-binding protein
LCN2/NGAL: Lipocalin 2
LPS: Lipopolysaccharide
MUC2: Mucin 2
NLRP: Nucleotide-binding oligomerization domain, leucine-rich repeat and pyrin
NG: Normoglycemia
NOD2: Nucleotide-binding oligomerization domain containing 2
OCLN1: Ocludin 1
PTL: Parthenolide
S100A: S100 Calcium-binding protein A
STEAP4: Six-transmembrane epithelial antigen of prostate 4
SPP1: Osteopontin
SVFC: Stroma-vascular fraction cells
TJP1/ZO1: Tight junction protein 1
TLR4: Toll-like receptor-4
TNF: Tumor necrosis factor-α
T2D: Type 2 diabetes
