# Supplementary material for: Decreased expression of the NLRP6 inflammasome is associated with increased intestinal permeability and inflammation in obesity with type 2 diabetes
Source: Cell Mol Life Sci. 2024 Feb 5;81(1):77. doi: 10.1007/s00018-024-05124-3 (PMC10844155; doi:10.1007/s00018-024-05124-3)
Supplement: Supplementary file 1 — Supplementary file1 (DOCX 565 KB) [file 18_2024_5124_MOESM1_ESM.docx]

**Supplemental Table 1.** Sequences of the primers and TaqMan^®^ probes.

| **Gene (GenBank accession)** | | **Oligonucleotide sequence (5’-3’)** | |
| --- | --- | --- | --- |
| *ADIPOQ* (NM_001177800) | |  | |
| Forward | | GGAGATCCAGGTCTTATTGGTCCTA | |
| Reverse | | CCTTGGATTCCCGGAAAGC | |
| TaqMan^®^ Probe | | FAM-ACATCGGTGAAACCGGAGTACCCGG-TAMRA | |
| *CCL2* (NM_002982) | |  | |
| Forward | | GCTCATAGCAGCCACCTTCATT | |
| Reverse | | TCTGCACTGAGATCTTCCTATTGGT | |
| TaqMan^®^ Probe | | FAM-TCGCTCAGCCAGATGCAATCAATGC-TAMRA | |
| *CLDN1* (NM_021101.5) | |  | |
| Forward | | AAAGTCTTTGACTCCTTGCTGAATCT | |
| Reverse | | TTCCAAGCACTTCATACACTTCATG | |
| TaqMan^®^ Probe | | FAM-AGCAGCCACATTGCAAGCAACCCGT-TAMRA | |
| *IL1B* (NM_000576) | |  | |
| Forward | | CAGTGGCAATGAGGATGACTTG | |
| Reverse | | GTAGTGGTGGTCGGAGATTCGTA | |
| TaqMan^®^ Probe | | FAM-TGGCCCTAAACAGATGAAGTGCTCCTTCC-TAMRA | |
| *IL18* (NM_001562) | |  | |
| Forward | | CCAAGGAAATCGGCCTCTATT | |
| Reverse | | CCTCTAGGCTGGCTATCTTTATACATACT | |
| TaqMan^®^ Probe | | FAM-TTCTGACTGTAGAGATAATGCACCCCGGAC-TAMRA | |
| *IL33* (NM_033439) | |  | |
| Forward | | CACTGAAAACAGGTAGAAAGCACAAA | |
| Reverse | | CCCCTGATATACCAAAGGCAAAG | |
| TaqMan^®^ Probe | | FAM-CTGGTACTCGCTGCCTGTCAACAGCAG-TAMRA | |
| *KLF4* (NM_001314052.1) | |  | |
| Forward | | ACCTACACAAAGAGTTCCCATCTCA | |
| Reverse | | GTTTACGGTAGTGCCTGGTCAGTT | |
| TaqMan^®^ Probe | | FAM-CTGCGAACCCACACAGGTGAGAAA-TAMRA | |
| *MUC2* (NM_002457) | |  | |
| Forward | | ACGGCCTGCAGAGCTATTCA | |
| Reverse | | TGATCTTCTGCATGTTCCCAAAC | |
| TaqMan^®^ Probe | | FAM-ATTCCTCTCTGACGGCGTGCTCTTCAGT-TAMRA | |
| *NGAL* (NM_005564) | |  | |
| Forward | | CCCAGCCCCACCTCTGA | |
| Reverse | | CTTCCCCTGGAATTGGTTGTC | |
| TaqMan^®^ Probe | | FAM-CAAGGTCCCTCTGCAGCAGAACTTCCA-TAMRA | |
| *NLRP1* (NM_033004.3) | |  | |
| Forward | | CCCTGGAGCAGGAGAAACCT | |
| Reverse | | CGAGTCTCTGCCGCTTGAGT | |
| TaqMan^®^ Probe | | FAM-CTCATCTTCAGCAGACGGAAACCAAGTGTG-TAMRA | |
| *NLRP3* (NM_001079821.2) | |  | |
| Forward | | AAGCTTCAGGTGTTGGAATTAGACA | |
| Reverse | | GTTGCCCAGGCTCAGCTTT | |
| TaqMan^®^ Probe | | FAM-CACACTGCTGCTGGGATCTTTCCACA-TAMRA | |
| *NLRP6* (NM_138329.2) | |  | |
| Forward | | TCCCTTCTTCATCCACTCTTTCAG | |
| Reverse | | CAGACCGCGTCAGGGAGTT | |
| TaqMan^®^ Probe | | FAM-CTGAGCAGCCTCACGCTGTCCCA-TAMRA | |
| *NOD2* (NM_001293557) | |  |  |
| Forward | | TTCAGGAATTACCAGTCCCATTG |  |
| Reverse | | GGTCCTCAGCTTGGCCATATACT |  |
| TaqMan^®^ Probe | | FAM-CCCTGCCTTTGGAAGCTGCCACA-TAMRA |  |
| *OCLN* (NM_001205254.2) | |  | |
| Forward | | TGGACTCTACGTGGATCAGTATTTGTA | |
| Reverse | | AGCAAAAGCCACAATAATCATGAAC | |
| TaqMan^®^ Probe | | FAM-TCCCCAGGAGGCCATTGCCAT-TAMRA | |
| *S100A8* (NM_002964) | |  | |
| Forward | | CGTCTACAGGGATGACCTGAAGA | |
| Reverse | | GACGTCTGCACCCTTTTTCCT | |
| TaqMan^®^ Probe | | FAM-TTGCTAGAGACCGAGTGTCCTCAGTAT-TAMRA | |
| *S100A9* (NM_002965) | |  | |
| Forward | | CTCAAGAAGGAGAATAAGAATGAAAAGG | |
| Reverse | | TCAGCTGCTTGTCTGCATTTG | |
| TaqMan^®^ Probe | | FAM-CATAGAACACATCATGGAGGACCTGGAC-TAMRA | |
| *SPP1* (NM_000582) | |  | |
| Forward | | CATCCAGTACCCTGATGCTACAGA | |
| Reverse | | GGCCTTGTATGCACCATTCAA | |
| TaqMan^®^ Probe | | FAM-ACATCACCTCACACATGGAAAGCGAGGA-TAMRA | |
| *STEAP4* (NM_024636) | |  | |
| Forward | | TCTCCAGTCAGGAGCACTGGAT | |
| Reverse | | CAATTTCTTTGGCTGCCATGA | |
| TaqMan^®^ Probe | | FAM-CAAGTCGGCAGGTGTTTGTGTGTGGA-TAMRA | |
| *TLR4* (NM_003266) | |  | |
| Forward | | CTGCGTGGAGGTGGTTCCTA | |
| Reverse | | CAGGTCCAGGTTCTTGGTTGAG | |
| TaqMan^®^ Probe | | FAM-TTTCTACAAAATCCCCGACAACCTCCCCT-TAMRA | |
| *TJP1* (NM_003257.5) | |  | |
| Forward | | GCACAGCAATGGAGGAAACAG | |
| Reverse | | TCTCGTCCACCAGATATTGCAAT | |
| TaqMan^®^ Probe | | FAM-ACAACATACAGTGACGCTTCACAGGGCTC-TAMRA | |
| *rNlrp1* (NM_001145755.2) | |  | |
| Forward | | CTGGAGACAAAGAATCCGAAGCT | |
| Reverse | | GCTCTCTTCTCCATCTGTGTTTTCA | |
| TaqMan^®^ Probe | | FAM-CACATGGATGTCACACATGACGATGCC-TAMRA | |
| *rNlrp3* (NM_001191642.1) | |  | |
| Forward | | GTTTTCCCAGACCCTCATGTTG | |
| Reverse | | AGAGACCTCGGCAGAAGCTAGAG | |
| TaqMan^®^ Probe | | FAM-CTTCCAGACTGGTGAACTGCTGCCTCA-TAMRA | |
| *rNlrp6* (NM_134375.3) | |  |  |
| Forward | | CCAAATGCAAGGTGCAGACA |  |
| Reverse | | CACAATGACCAGGTAGTGGATCAC |  |
| TaqMan^®^ Probe | | FAM-CAGGATACAGATGCCTGGCCTCCAAGA-TAMRA |  |

*ADIPOQ,* adiponectin; *CCL2*, monocyte chemoattractant protein-1; *CLDN1*, claudin 1; *IL*, interleukin; *KLF4*, kruppel like factor 4; *MUC2*, mucin 2; *NGAL*, lipocalin 2; *NLRP*, nucleotide-binding oligomerization domain, leucine rich repeat and pyrin; *NOD2*, nucleotide binding oligomerization domain containing 2; *OCLN*, occludin; *S100A9*, S100 calcium-binding A9; *SPP1*, osteopontin; *STEAP4*, STEAP4 metalloreductase; *TLR4*, toll-like receptor-4; *TJP1*, tight junction protein 1.

**Supplemental Table 2**. Univariate analysis of the correlations between circulating levels of intestinal dysfunction markers and anthropometric and metabolic variables.

|  | **Endotoxin** | | **LBP** | | **Zonulin** | | **Lactoferrin** | | **S100A8** | | **CCL5** | | **mRNA *NLRP6*** | | **mRNA *IL18*** | |
| --- | --- | --- | --- | --- | --- | --- | --- | --- | --- | --- | --- | --- | --- | --- | --- | --- |
|  | r | *P* | r | *P* | r | *P* | r | *P* | r | *P* | r | *P* | r | *P* | r | *P* |
| **Endotoxin** | - | - | 0.11 | 0.336 | 0.23 | **0.041** | 0.31 | **0.008** | 0.11 | 0.339 | 0.38 | **<0.001** | -0.68 | **0.044** | 0.11 | 0.742 |
| **LBP** | 0.11 | 0.336 | - | - | 0.37 | **<0.001** | 0.44 | **<0.001** | 0.33 | **0.003** | 0.33 | **0.003** | -0.77 | **0.015** | 0.29 | 0.359 |
| **Zonulin** | 0.23 | **0.041** | 0.37 | **<0.001** | - | - | 0.21 | 0.073 | 0.25 | **0.028** | 0.17 | 0.153 | -0.58 | 0.103 | 0.03 | 0.908 |
| **Lactoferrin** | 0.31 | **0.008** | 0.44 | **<0.001** | 0.21 | 0.073 | - | - | 0.60 | **<0.001** | 0.29 | **0.011** | 0.23 | 0.548 | -0.35 | 0.269 |
| **S100A8** | 0.11 | 0.339 | 0.33 | **0.003** | 0.25 | **0.028** | 0.60 | **<0.001** | - | - | 0.56 | **<0.001** | -0.44 | 0.179 | -0.11 | 0.733 |
| **CCL5** | 0.38 | **<0.001** | 0.31 | **0.007** | 0.17 | 0.153 | 0.29 | **0.011** | 0.56 | **<0.001** | - | - | **0.78** | **0.014** | 0.67 | **0.033** |
| **mRNA *NLRP6*** | -0.68 | **0.044** | -0.77 | **0.015** | -0.58 | 0.103 | 0.23 | 0.548 | -0.44 | 0.179 | 0.57 | 0.109 | - | - | 0.79 | **<0.001** |
| **mRNA *IL18*** | 0.11 | 0.742 | 0.29 | 0.359 | 0.03 | 0.908 | -0.35 | 0.269 | -0.11 | 0.733 | 0.67 | **0.033** | 0.79 | **<0.001** | - | - |
| **BMI** | 0.11 | 0.350 | 0.44 | **<0.001** | 0.24 | **0.039** | 0.46 | **<0.001** | 0.51 | **<0.001** | 0.13 | 0.281 | -0.23 | 0.449 | 0.14 | 0.644 |
| **Body fat** | 0.13 | 0.273 | 0.43 | **<0.001** | 0.26 | **0.029** | 0.32 | **0.006** | 0.48 | **<0.001** | 0.02 | 0.852 | 0.16 | 0.610 | 0.33 | 0.257 |
| **WHtR** | 0.03 | 0.790 | 0.37 | **<0.01** | 0.29 | **0.014** | 0.36 | **0.002** | 0.41 | **<0.001** | 0.01 | 0.899 | -0.15 | 0.625 | 0.08 | 0.785 |
| **Insulin** | 0.30 | **0.018** | 0.25 | **0.045** | 0.20 | 0.124 | 0.12 | 0.322 | 0.13 | 0.288 | 0.06 | 0.598 | -0.78 | **0.003** | -0.68 | **0.011** |
| **HOMA** | 0.25 | **0.042** | 0.25 | **0.047** | 0.45 | **<0.001** | 0.16 | 0.192 | 0.21 | 0.101 | 0.10 | 0.405 | -0.59 | **0.046** | -0.64 | **0.018** |
| **QUICKI** | -0.21 | 0.078 | -0.31 | **0.013** | -0.37 | **0.002** | -0.13 | 0.317 | -0.26 | **0.036** | 0.11 | 0.396 | 0.44 | 0.157 | 0.63 | **0.021** |
| **HDL-cholesterol** | -0.14 | 0.282 | -0.33 | **0.009** | -0.33 | **0.009** | -0.34 | **0.006** | -0.35 | **0.005** | -0.11 | 0.381 | 0.26 | 0.399 | 0.05 | 0.862 |
| **AST/ALT** | 0.12 | 0.376 | -0.22 | 0.092 | -0.26 | **0.044** | -0.27 | **0.037** | -0.49 | **<0.001** | -0.13 | 0.333 | -0.21 | 0.486 | 0.01 | 0.998 |

AST/ALT, aspartate aminotransferase/alanine aminotransferase ratio; BMI, body mass index; CCL5/RANTES, C-C motif chemokine ligand 5; HOMA, homeostatic model assessment; IL, interleukin; LBP, lipopolysaccharide binding protein; *NLRP*, nucleotide-binding oligomerization domain, leucine rich repeat and pyrin; QUICKI, quantitative insulin sensitivity check index; S100A8, S100 calcium-binding protein A8; WHtR, waist-to-height ratio

**Supplemental Table 3**. Effects of weight loss in patients with obesity after Roux-en-Y gastric bypass and conventional diet.

|  | **RYGB** | | **Conventional diet** | |
| --- | --- | --- | --- | --- |
|  | **Before WL** | **After WL** | **Before WL** | **After WL** |
| n (male, female) | 20 (8, 12) | 20 (8, 12) | 20 (12, 8) | 20 (12, 8) |
| Age (years) | 45 ± 3 | 46 ± 3 | 47 ± 4 | 48 ± 4 |
| BMI (kg/m^2^) | 44.2 ± 1.2 | 29.3 ± 0.8^***^ | 33.8 ± 1.2 | 29.2 ± 1.3^***^ |
| Body fat (%) | 51.1 ± 0.9 | 34.9 ± 1.3^***^ | 42.8 ± 1.9 | 36.6 ± 2.1^***^ |
| Waist circumference (cm) | 126 ± 1 | 96 ± 3^***^ | 109 ± 4 | 98 ± 3^***^ |
| Fasting glucose (mg/dL) | 111 ± 8.2 | 92 ± 3^**^ | 95 ± 3 | 90 ± 2 |
| Fasting insulin (μU/mL) | 19.4 ± 3.0 | 6.4 ± 1.4^**^ | 11.9 ± 1.1 | 9.6 ± 1.7 |
| HOMA | 5.6 ± 0.3 | 1.8 ± 0.5^***^ | 2.5 ± 0.6 | 2.2 ± 0.9 |
| QUICKI | 0.315 ± 0.034 | 0.393 ± 0.052^**^ | 0.343 ± 0.038 | 0.355 ± 0.049 |
| Triglycerides (mg/dL) | 130 ± 18 | 90 ± 9^**^ | 121 ± 13 | 83 ± 6^**^ |
| Cholesterol (mg/dL) | 200 ± 5 | 168 ± 3^***^ | 201 ± 10 | 175 ± 8^*^ |
| LDL-cholesterol (mg/dL) | 124 ± 3 | 90 ± 1^**^ | 122 ± 10 | 109 ± 6 |
| HDL-cholesterol (mg/dL) | 50 ± 4 | 61 ± 5^***^ | 55 ± 3 | 51 ± 2 |

BMI, body mass index; HOMA, homeostatic model assessment; QUICKI, quantitative insulin sensitivity check index; RYGB, Roux-en-Y gastric bypass; WL, weight loss. Data are mean ± SEM. Differences between groups were analyzed by paired two-tailed Student’s *t* tests. ^*^*P*<0.05, ^**^*P*<0.01 and ^***^*P*<0.001 *vs* before WL.


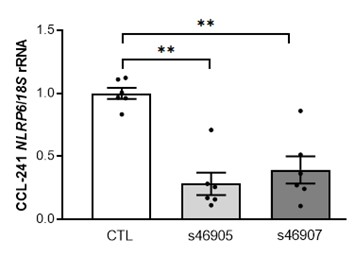


**Supplemental Fig. 1**. Gene expression levels of *NLRP6* in human CCL-241 cell line after transfection with two pairs of siRNAs (s46905 and s46907) at 200 pmol/L siRNA *NLRP6*/2x10^5^ cells for 24 h. Gene expression levels in scrambled siRNA cells (CTL) were assumed to be 1. Values are the mean ± SEM (n=6 per group). Differences between groups were analyzed by unpaired two-tailed Student’s *t* tests. ^**^*P*<0.01 *vs* CTL.

**
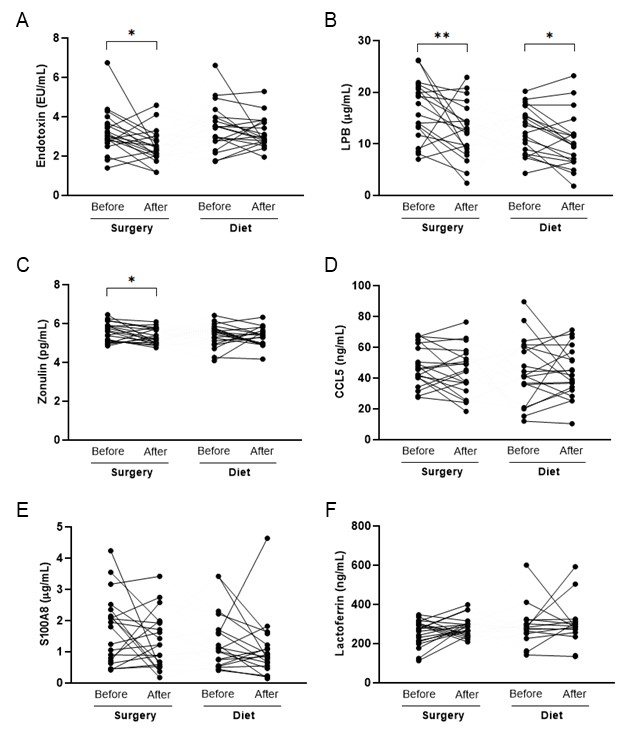
**

**Supplemental Fig. 2**. Circulating levels of A) endotoxin, B), lipopolysaccharide binding protein (LBP), C) zonulin, D) C-C motif chemokine ligand 5 (CCL5/RANTES), E) S100 calcium-binding protein A8/calprotectin A (S100A8) and F) lactoferrin before and after weight loss achieved by Roux-en-Y gastric bypass (RYGB) (n=20) or a conventional diet (n=20). Differences between groups were analysed by paired Student’s *t*-test. ^*^*P*<0.05 and ^**^*P*<0.01.

**
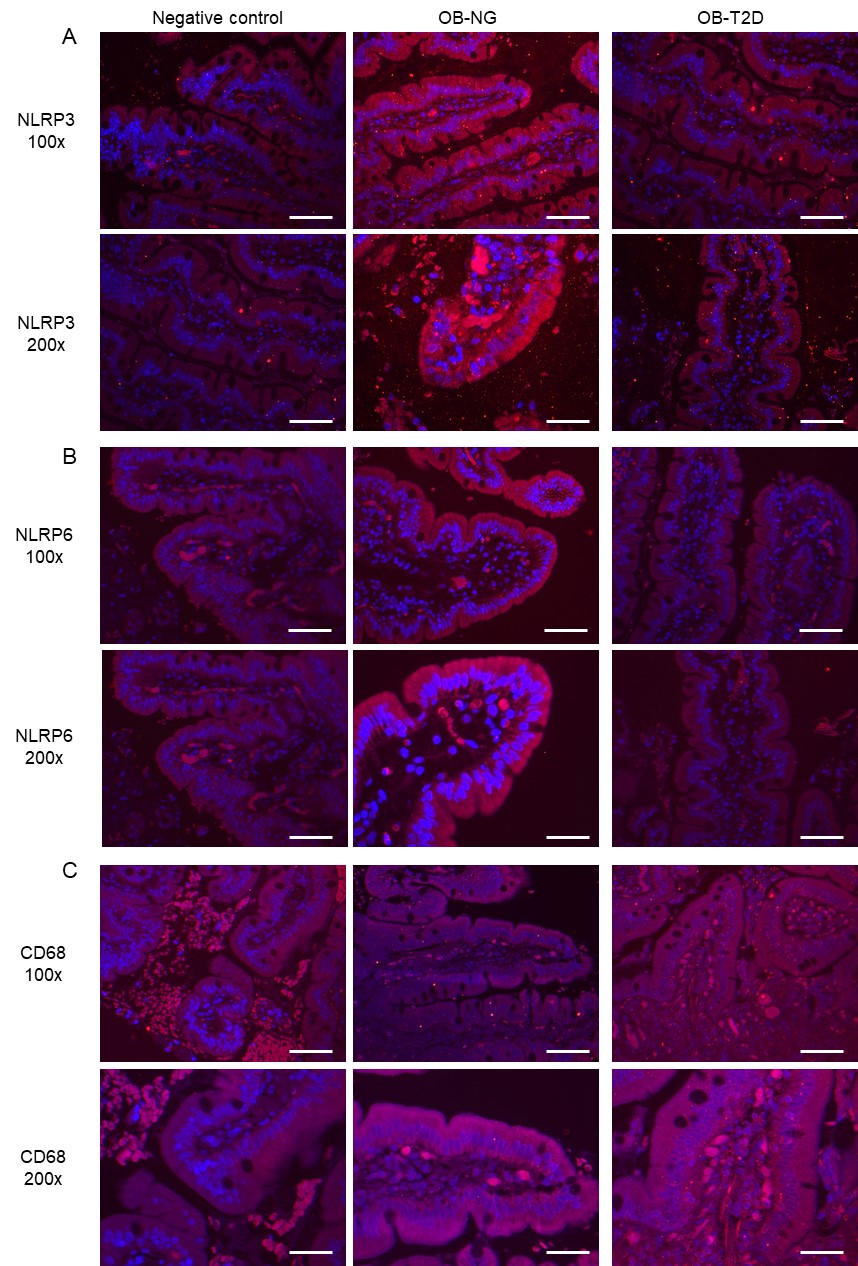
Supplemental Fig. 3**. Immunofluorescence detection of (A) NLRP3, (B) NLRP6 and (C) CD68 in histological sections of human jejumun. Representative images of at least 3 separate experiments are shown [scale bar (100x: 50 μm; 200x: 100 μm)].


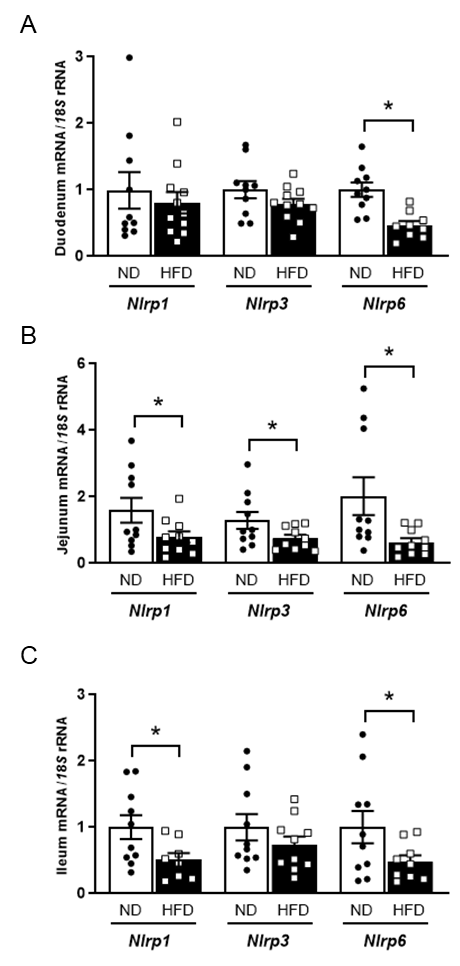


**Supplemental Fig. 4**. Gene expression levels of *Nlrp1*, *Nlrp3* and *Nlrp6* in the (A) duodenum, (C) jejunum and (E) ileum from rats fed a normal diet (ND) or a high-fat diet (HFD). Unpaired Student’s t-test was used to calculate differences between ND and HFD groups. **P*<0.05. *Nlrp*, nucleotide-binding oligomerization domain, leucine rich repeat and pyrin.


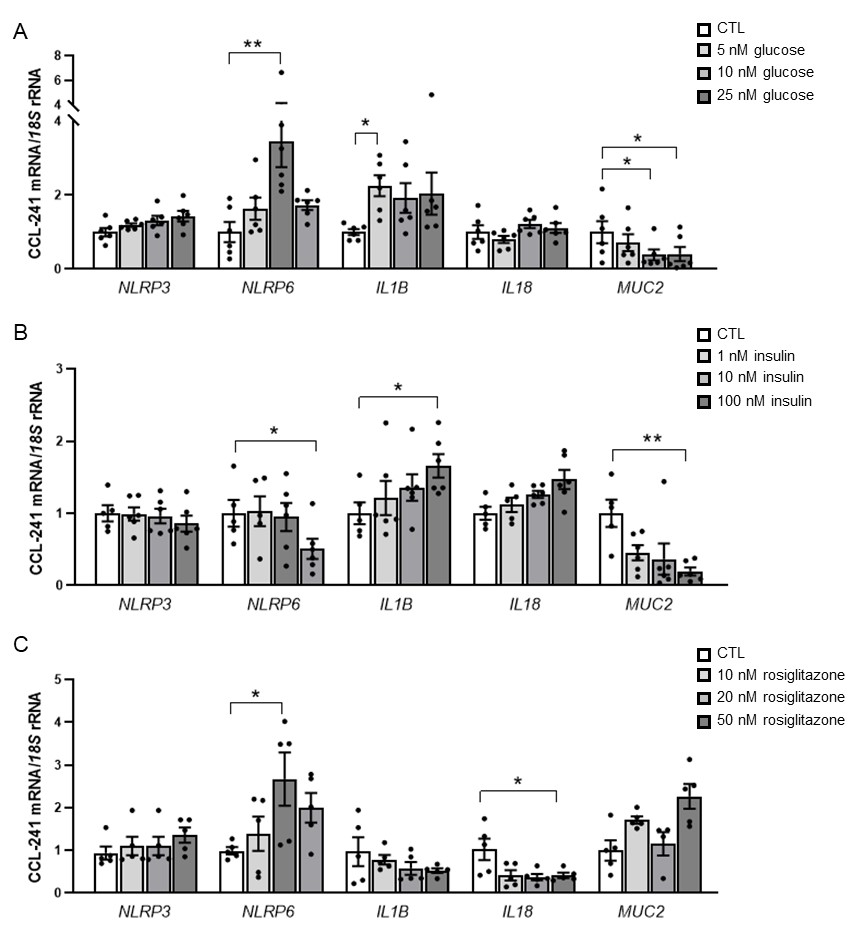


**Supplemental Fig 5.** Gene expression levels of the inflammasome components (*NLRP3* and *NLRP6*), their main effectors (*IL1B* and *IL18*) and the intestinal integrity genes (*MUC2* and *TJP1*) in human enterocytes CCL-241 cells treated with different concentrations of (A) glucose, (B) insulin and (C) rosiglitazone during 24 h (n=6 per group). Values are the mean ± SEM (n=6 per group). Differences between groups were analyzed by one-way ANOVA followed by Dunnett’s tests. ^*^*P*<0.05 and ^**^*P*<0.01 *vs* unstimulated cells. *NLRP*, nucleotide-binding oligomerization domain, leucine rich repeat and pyrin; *IL*, interleukin; *MUC2*, mucin 2.
